# In vitro evaluation of the combination between some antibiotics and essential oils of clove and rosemary against Pseudomonas aeruginosa

**DOI:** 10.1186/2047-2994-4-S1-P169

**Published:** 2015-06-16

**Authors:** L El Hosseiny, M El-Shenawy

**Affiliations:** 1 Department of Environmental Studies, Institute of Graduate Studies and Research, Alexandria University, Alexandria, Egypt; 2 Department of Food & Environmental Microbiology, National Research Center, Dokki, Cairo, Egypt

## Introduction

Emergence of resistance to multiple antimicrobial agents threatens public health at a global scale. One of the most clinically significant multidrug resistant pathogens is Pseudomonas aeruginosa.

## Objectives

The association of antibiotics and plant extracts against resistant pathogens is one of the promising choices for the treatment of infectious diseases.

## Methods

This work addresses the antibiotic enhancing effect of two essential oils against Pseudomonas aeruginosa. Clove and rosemary essential oils were hydrodistilled from the buds of Syzygiumaromaticum and leaves of and Rosmarinusofficinalis respectively. The chemical composition of the extracted essential oils was identified using gas chromatography coupled mass spectrospcopy analysis. Disc diffusion assay was used to investigate the antibacterial activity of clove and rosemary essential oils against Pseudomonas aeruginosa (ATCC 9027). Furthermore, the antibiotic enhancement capacity of these oils was evaluated in combination with some antipseudomonal drugs comprising ceftazidime, imipenem, aztreonam and ciprofloxacin.

## Results

Results revealed that clove essential oil exhibited higher activity towards the test bacterium than rosemary oil. Meanwhile, the antipseudomonal activities of all the tested antibiotics were enhanced in the range of 8-50% when combined with clove oil and 12-33.3% in case of rosemary oil. The antibacterial activity displayed by both essential oils, alone and in association with the antibiotics, is probably related to the major components identified in both oils, comprising eugenol (80.03%) in clove and eucalyptol (29.3%) in rosemary.

## Conclusion

Clove and rosemary essential oils are potential candidate antimicrobial natural products that could enhance the activity of conventional antibiotics.

## Disclosure of interest

None declared.

